# P-1805. Identifying Hospital Antimicrobial Usage Trends Using Wastewater Based Surveillance

**DOI:** 10.1093/ofid/ofae631.1968

**Published:** 2025-01-29

**Authors:** Matthew Penney, Darina Kuzma, Aleshia Kormendi, Kayla Moffett, Christine O’Grady, Rhonda Clark, Diego Nobrega, Susana Kimura-Hara, Janine McCalder, Laura Vivas, Chloe Papparis, Nicole Acosta, Barbara Jean M Waddell, Casey R J Hubert, Bruce Dalton, John Conly, Michael Parkins, Benson Weyant, Elissa Rennert-May

**Affiliations:** The University of Calgary, Calgary, Alberta, Canada; The University of Calgary, Calgary, Alberta, Canada; The University of Calgary, Calgary, Alberta, Canada; The University of Calgary, Calgary, Alberta, Canada; University of Calgary, Calgary, Alberta, Canada; University of Calgary, Calgary, Alberta, Canada; The University of Calgary, Calgary, Alberta, Canada; The University of Calgary, Calgary, Alberta, Canada; University of Calgary, Calgary, Alberta, Canada; University of Calgary, Calgary, Alberta, Canada; University of Calgary, Calgary, Alberta, Canada; University of Calgary, Calgary, Alberta, Canada; University of Calgary, Calgary, Alberta, Canada; University of Calgary, Calgary, Alberta, Canada; Alberta Health Services, Calgary, Alberta, Canada; University of Calgary, Calgary, Alberta, Canada; University of Calgary, Calgary, Alberta, Canada; University of Calgary, Calgary, Alberta, Canada; University of Calgary, Calgary, Alberta, Canada

## Abstract

**Background:**

Hospitals represent ideal locations for developing wastewater (WW) surveillance for antibiotics (Abx), owing to the high frequency of Abx use and robust record-keeping. To investigate this technology as a potentially useful stewardship tool, we compared the concentration of several Abx in WW from tertiary care hospitals to their corresponding levels in the surrounding municipality.

Concentration of 4 common antimicrobials found in hospital wastewater effluent over a 4-month period

**Figure:**
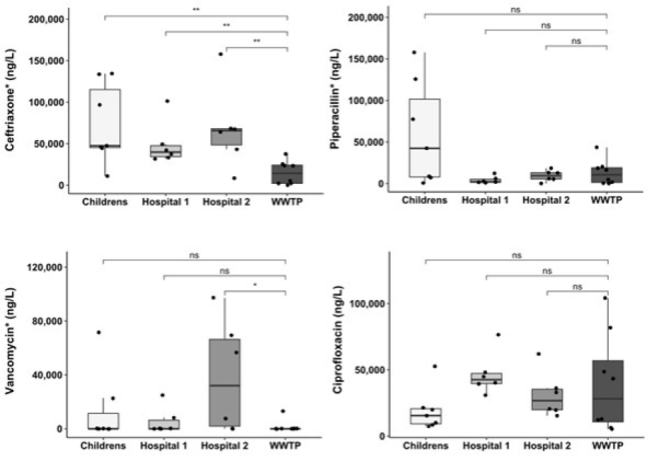

Concentrations of 4 antimicrobials measured in WW from 3 tertiary care hospitals and the corresponding wastewater treatment plant (WWTP) in the same city. Displayed statistics represent the results of a Wilcoxon test, antimicrobials labelled “*” are commonly administered intravenously.

**Methods:**

WW was collected bi-weekly from three tertiary care hospitals (two adult and one pediatric, with 600, 650 and 135 inpatient beds, respectively) and the associated municipal WW treatment plant (serving a population of ∼1,000,000) between February and May of 2024. Aliquots of WW were filtered and run directly on a liquid-chromatography paired triple quadrupole mass spectrometer (LC-QQQ) to quantify specific Abx (azithromycin, doxycycline, ciprofloxacin, levofloxacin, metronidazole, cefazolin, ceftriaxone, piperacillin, tazobactam, meropenem, vancomycin, and sulfamethoxazole). Spiked and replicate samples were randomly included to validate analyte recovery and reproducibility. Agilent MassHunter software (Version 10.1, 2019) was utilized to process and export raw data to R. Box plots and Wilcoxon tests were utilized to compare the concentration of each Abx.

**Results:**

Validation experiments confirmed that filtering WW samples and directly running them on LC-QQQ yields reproducible and reliable results. Serial monitoring revealed that WW from hospitals generally exhibited a broader range of Abx concentrations than was observed city-wide (Figure 1). This variability was particularly evident among the most used Abx, consistent with the changing treatment needs of highly dynamic hospital populations. In addition to this, ceftriaxone, an IV-administered antimicrobial was found at significantly higher concentrations in all hospital sites when compared to the municipal WW treatment plant.

**Conclusion:**

Validating Abx monitoring in WW from a range of scales will enable this approach to be applied across diverse environments as a tool to mitigate Abx resistance. This approach will be strengthened as it is integrated with clinical metadata and metagenomic assessment of antimicrobial resistance genes from the same samples.

**Disclosures:**

**All Authors**: No reported disclosures

